# A Review of How Lasers Are Used in UTUC Surgery: Can the Choice of Laser Affect Outcomes?

**DOI:** 10.3390/cancers15061874

**Published:** 2023-03-21

**Authors:** Mark Taratkin, Nirmish Singla, Diana Babaevskaya, Alexander Androsov, Shahrokh F. Shariat, Harun Fajkovic, Jack Baniel, Dmitry Enikeev

**Affiliations:** 1 Institute for Urology and Reproductive Health, Sechenov University, 119435 Moscow, Russia; 2 Department of Urology, James Buchanan Brady Urological Institute, Johns Hopkins University School of Medicine, Baltimore, MD 21287, USA; 3 Department of Oncology, Johns Hopkins University School of Medicine, Baltimore, MD 21287, USA; 4 Institute for Clinical Medicine, Sechenov University, 119021 Moscow, Russia; 5 Department of Urology, Medical University of Vienna, 1030 Vienna, Austria; 6 Department of Urology, Weill Cornell Medical College, New York, NY 10065, USA; 7 Department of Urology, University of Texas Southwestern, Dallas, TX 75390, USA; 8 Division of Urology, Department of Special Surgery, Hourani Center for Applied Scientific Research, Al-Ahliyya Amman University, Amman 19328, Jordan; 9 Department of Urology, Second Faculty of Medicine, Charles University, 150 06 Prague, Czech Republic; 10 Karl Landsteiner Institute of Urology and Andrology, 3100 Vienna, Austria; 11 Division of Urology, Rabin Medical Center, Petach Tikva 4920232, Israel; 12 Sackler Faculty of Medicine, Tel Aviv University, Tel Aviv 6997801, Israel

**Keywords:** upper tract urothelial cell carcinoma (UTUC), laser ablation, kidney-sparing surgery, endoscopic treatment

## Abstract

**Simple Summary:**

Kidney-sparing surgery (KSS) and particularly endoscopic, has gained attractivity for the treatment of localized upper tract urothelial carcinoma (UTUC). Endoscopy results in satisfactory oncological disease control while lowering morbidity and minimizing complications in appropriately selected patients. However, up to now, there is still no solid data as to which laser energy source is superior to the others. The goal of this review is to outline the results of endoscopic UTUC treatment using different lasers and to analyze how these laser-tissue interactions may affect the surgery. Herein, we report that data remains insufficient to support the superiority of one laser type over another in the endoscopic management of UTUC. The ever-growing number of indications for minimally invasive treatment and the increasing number of centers using laser surgery will, hopefully, lead to novel randomized controlled trials comparing the performance characteristics of the lasers as well as the effects on UTUC in patients.

**Abstract:**

Upper tract urothelial carcinoma (UTUC) is a relatively rare disease with an aggressive phenotype compared to urothelial carcinoma in the bladder. In recent years, kidney-sparing surgery (KSS) and, in particular, endoscopic surgery have become the procedure of choice among urologists where the treatment of localized UTUC is concerned. Endoscopy tends to result in satisfactory oncological disease control while lowering morbidity and minimizing complications amongst the appropriately selected cohort of patients. While endoscopic surgery for UTUC might appear to be standardized, it, in fact, differs considerably depending on the source of energy used for resection/ablation. There has been little reliable data up to now on which laser energy source is the most superior. The goal of this review is, therefore, to outline the results of endoscopic UTUC treatment using different lasers and to analyze how these laser-tissue interactions may affect the surgery. We start by pointing out that the data remains insufficient when trying to determine which laser is the most effective in the endoscopic management of UTUC. The ever-growing number of indications for minimally invasive treatment and the increasing number of centers using laser surgery will, hopefully, lead to novel randomized controlled trials that compare the performance characteristics of the lasers as well as the effects of UTUC on patients.

## 1. Introduction—UTUC and Kidney-Sparing Treatment Approaches

Kidney-sparing surgery (KSS) in upper tract urothelial carcinoma (UTUC) is now recommended by EAU and NCCN not only for patients with serious indications such as solitary kidneys, renal insufficiency or bilateral tumors but also for all patients with low-risk UTUC irrespective of the status of the contralateral kidney [1,2]. The EAU panel defines low-risk UTUC as unifocal, small (<2 cm), negative for high-grade cytology with low-grade URS biopsy and no invasive aspect on CT [3]. These recommendations have subsequently been expanded since the first guidelines on UTUC were published in 2015. At the time, these guidelines recommended that only tumors less than 1 cm with a functional contralateral kidney should be treated conservatively. The NCCN criteria of tumors that might be treated with KSS are stricter and include papillary, unifocal, low-grade tumors less than 1.5 cm in size, with no evidence of invasive disease on cross-sectional imaging [4]. Given the expanding indications, the number of trials on endoscopic management (KSS) of low-risk UTUC has increased over the last decade [5,6].

Most of the KSS studies in UTUC focus on the type of energy for endoscopic UTUC management. Recent data even promises to expand the indications for KSS supporting endoscopic management in large (>2 cm) and multifocal tumors [5,7,8,9]. In this review, we aim to discuss the current status of endoscopic laser surgery for UTUC, focusing on the physical aspects of each device, specifically by systemizing the data on the pros and cons of available energy sources.

## 2. Materials and Methods

A systematic literature search using two databases (Medline (PubMed) and Scopus) was performed. 

**Figure 1:**
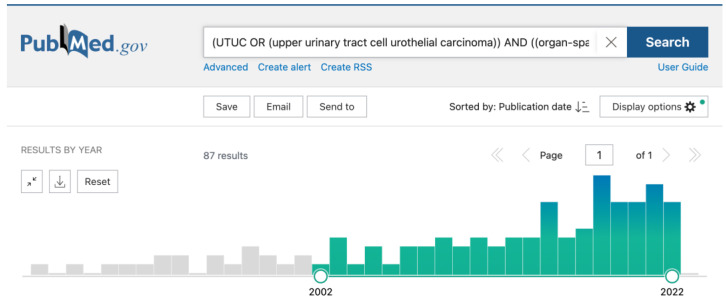
Dynamics of publications on the topic over the past 20 years.

The following search query was used: “(UTUC OR (upper urinary tract cell urothelial carcinoma)) AND ((organ-sparing (treatment OR surgery) OR kidney sparing OR kidney-sparing OR KSS OR renal sparing OR renal-sparing OR endoscopic management OR ureteroscopic OR endoscopic treatment OR resection OR partial OR localized OR unifocal) AND (laser* OR Nd:YAG OR Ho:YAG OR Greenlaser OR Greenlight OR Tm:YAG OR TFL).” The dynamics of publications on this topic over the past 20 years are presented in Figure 1.

The inclusion criteria were as follows: all types of studies (both prospective and retrospective) containing their own data on outcomes of laser ablation surgery. We included only articles that were published in English. All articles that did not contain original data were also excluded, e.g., reviews, comments, single cases, editorial material, books or conference abstracts.

Firstly, DB and AA performed a title review. Secondly, they independently performed abstract reviews according to the same criteria. Once the titles and abstracts were reviewed, DB and AA manually removed any duplicates. In addition, all articles about different aspects of UTUC diagnostics, such as confocal laser endomicroscopy, about electrocautery endoscopic treatment, and about endoscopic treatment complications, were excluded. In addition, we excluded all articles which did not specify which laser system was used. As a last step, DB and AA independently performed a full-text review. In the event of any disagreement, each party made its case and tried to resolve it. If they could not come to an agreement, MT, who is an experienced urologist, made the final decision. The final review included 33 articles.

## 3. Results

### 3.1. Types of Conservative Treatment of UTUC

KSS includes endourological procedures, partial ureteral resection and total ureterectomy [2]. As technologies advance, endourological procedures (ureteroscopic and percutaneous approaches) are becoming preferable among urologists due to their minimal invasiveness and low complexity. The EAU Non-muscle Invasive Bladder Cancer Guidelines Panel suggests that endourological treatment has a cancer-specific and overall survival rate comparable to radical nephroureterectomy for correctly selected patients [3]. The percutaneous approach is less preferred compared with URS in treating UTUC due to the slightly higher perioperative morbidity [4]. Cutress et al., in their systematic review, pointed out that the percutaneous approach could be an option for tumors not accessible ureteroscopically, while the theoretical risk for tumor seeding in the nephrostomy route remains questionable [4]. In cases of disease recurrence, a history of endoscopic management does not increase the risks and does not affect survival after subsequent radical nephroureterectomy (RNU) [10]. In general, endourological procedures in low-grade UTUC show similar oncological outcomes (overall survival and cancer-specific survival) compared to the radical treatment [11]. However, they potentially increase the risk of intravesical seeding and recurrence.

### 3.2. Nd:YAG

The first laser used in the conservative management of UTUC was a neodymium-doped yttrium aluminum garnet laser (Nd:YAG), which is rarely used nowadays [12]. It was one of the first laser devices in surgery and used hemoglobin as a target chromophore (a substance that absorbs laser energy). This resulted in a rather profound depth of penetration (up to 10 mm) compared to other lasers (e.g., Ho:YAG, which has a penetration depth of 0.4–0.7 mm). Whilst operating in the no-contact mode, vaporization by the Nd:YAG laser causes larger tissue defects and coagulation necrosis depending on the time and dose, while Ho:YAG laser application leads to tissue ablation without any relevant coagulation necrosis [13].

These laser features allow for the vaporization and deep coagulation of large and bulky tumors [14]. The physical properties of Nd:YAG laser can, however, lead to high rates of ureteral perforation, and thus it should be used preferably for tumors located in the renal pelvicalyceal system [15]. In a study by Scotland et al., both Ho:YAG and Nd:YAG lasers have resulted in a PFS rate of 75.18% at 5 years of follow-up [5]. Similarly, Niţă et al. found a recurrence rate for low-grade tumors after Nd:YAG treatment was 36.36% (16 out of 44 cases) during the mean follow-up period of 60 months [16]. 

The earlier studies show comparable results. Grossman et al. [17] also tried to estimate the oncological outcomes after Nd:YAG ureteroscopic tumor ablation. They included eight patients in their study; two of those underwent radical surgery after endoscopic treatment (one because of tumor progression and one due to technical reasons). Of the remaining six patients, one patient refused the follow-up evaluation; two patients had no recurrences after the primary intervention; one patient after two recurrences was treated successfully and was tumor-free for 21 months; and two patients continued to show active disease rates and were managed endoscopically without evidence of tumor progression. 

The earlier study of Orihuela et al. [18], published in 1988, produced similar results: only six out of the 14 treated patients were free of recurrence at a mean of 19 months follow-up. More favorable results were achieved by Kaufman et al. in 1993 [19]. In that study, eight out of nine highly selected patients with low-grade, low-stage transitional cell carcinoma of the ureter had no evidence of disease recurrence at a mean of 28 months of follow-up. In the article by Malloy et al. from 1986 [20], the same results were reported: six patients treated using Nd:YAG did not have any recurrence at a mean follow-up of 14 months after surgery.

The comparison between Nd:YAG and electrocoagulation was performed by Elliott et al. in the 90’s [21]. The results for both types of ablations were approximately the same: 44 patients underwent laser ablation, 50.0% of them had at least one recurrence, and 50.4% of patients had recurrences after electrocoagulation. Later, in 2001 Elliott et al. [22] published the results of patients separately without strict indications for KSS. In that group, six out of eight patients (75%) after Nd:YAG ablation had no recurrence at a mean follow-up of 74 months. 

To sum up, even the most recent studies on Nd:YAG were performed before 2015 and were retrospective. This is mostly due to the above-mentioned technical limitations, together with a growing number of better energy sources that have supplanted Nd:YAG as a laser energy source in the management of UTUC.

### 3.3. Ho:YAG

One of the most commonly used lasers in the urological field is the holmium:yttrium-aluminium-garnet laser (Ho:YAG), which also plays a major role in the treatment of UTUC. This is due, in large part, to its technical characteristics: its wavelength is highly absorbed in water [23], which means the penetration depth can be decreased to 0.4–0.7 mm [24]. Ho:YAG is a pulsed laser with a significant peak power resulting in significant vapor generation during firing, resulting in ruptured incisions with sufficient coagulation properties [24]. 

Being an effective cutting device, Ho:YAG can be used both for tumor resection and vaporization. However, the above-mentioned effect of tissue rupture may limit the utility of the device [23]. Recent reports suggest that novel holmium lasers with pulse modulation may result in enhanced tissue cutting and coagulation [25]. However, no clinical data on pulse modulation in UTUC treatment is available at the moment.

The oncological outcomes of Ho:YAG seems to be at least satisfactory. Cornu et al. performed an analysis of 35 patients who were treated by ureteroscopy using Ho:YAG laser; 15 of the patients had an imperative indication for conservative treatment. At the 3-year follow-up, the disease-specific survival was 100%, and the recurrence-free survival was 35% [26]. In the large patient series reported on by Villa et al., the photoablation with Ho:YAG resulted in a PFS rate of 72% for patients with low-risk cancer after a median follow-up of 52 months [9]. Hoffman et al. performed a case-control study comparing URS with Ho:YAG ablation and radical surgery. During a median follow-up of 26 months, no disease-related morbidity was found in the group of patients treated with laser ablation, but, as expected, the bladder recurrence rate was higher than that in patients after RNU (44% vs. 39%, respectively) [27].

Painter et al. [28] found in their study: 17 out of 19 patients with a normal contralateral kidney, normal global renal function, no major comorbidity, and favorable tumor characteristics determined by biopsy (smaller lesions, all grade 2 or less) who underwent tumor ablation using Ho:YAG laser showed no evidence of disease progression after the median surveillance of 24 months. At the same time, 12 out of 16 patients with less favorable tumor characteristics or moderate global chronic renal dysfunction or who refused radical surgery on a contralateral side previously had undergone a radical nephroureterectomy performed between 1 and 24 months from the ureteroscopic treatment. 

Yamane et al. [29] published two case reports that confirmed favorable outcomes of low-grade tumors. In the first case, an 82-year-old man with solitary kidney and low-grade urothelial carcinoma (pTaN0M0) showed no evidence of recurrence 7 years after surgery. A 67-yo woman with the same tumor characteristics that were managed using the same protocol as in Case 1 showed no evidence of recurrence 5 years after surgery. 

In the study of Matsuoka et al. [30], six out of seven (86%) patients with a single kidney had at least 1 recurrence during the observation period, so the tumor-free rate was only 57% (4/7) at the mean follow-up of 37 months. In the elective group (with two kidneys), only four out of 20 (20%) patients had a recurrence, with a tumor-free rate of 95% (19/20) at 33 months after surgery. The authors concluded that patients for whom laser ablation is an elective procedure demonstrate more favorable results compared to those for whom it is an imperative option (in most cases, these patients underwent the contralateral RNU previously).

The effect of combined endoscopic holmium surgery + adjuvant chemotherapy on outcomes is also of great interest. In the study by Aboumarzouk et al. [31], which included 19 patients who underwent Ho:YAG laser tumor ablation with the single postoperative instillation of Mitomycin C, 13 out of 19 patients remained cancer-free at a mean follow-up of 24 months. Of patients who developed recurrence, three suffered from an advanced disease at the G3pT1 stage and had a history of previously removed contralateral kidney or bladder. In the other four cases of recurrence, patients underwent repeated treatment with laser ablation and Mitomycin single instillation, and they remained tumor-free on subsequent follow-ups.

### 3.4. Tm:YAG

An ex vivo study by Proietti et al. found that thulium: yttrium-aluminium-garnet laser (Tm:YAG) is potentially safer and more efficient for UTUC compared with Ho:YAG [32]. Tm:YAG firing is absorbed by water in tissue (similar to Ho:YAG), which results in a low coagulation depth of 0.2–0.4 mm. Yet, unlike holmium lasers, Tm:YAG is a laser with a continuous firing that results in the same ablation/resection efficiency that can be maintained throughout the treatment procedure providing a shallow and precise tissue cutting. In the clinical setting, Tm:YAG resulted in a low rate of intraoperative complications, bleeding and perforation of the ureter [33]. However, these characteristics, as well as the continuous mode of Tm:YAG, lead to high carbonization, which may alter its histologic appearance and impact pathologic interpretation. In the context of tissue ablation, this aspect does not pose a limitation, but it should be taken into consideration. 

In a single-center study, Musi et al. described the outcomes of 42 patients who were treated using Tm:YAG. In that group, the rate of upper tract recurrence was 19%, with only 9.5% of cases progressing to RNU [34]. Wen et al. found that both the local and bladder recurrence rate after Tm:YAG treatment was low, yet worse than after RNU (21.9 and 13.1%, respectively, *p* < 0.01) [35]. However, the exact median length of follow-up was not stated in the paper. The comparative study of Tm:YAG versus Ho:YAG in ureteroscopic management of UTUC showed better performance scores for Tm:YAG as assessed by surgeons. However, there were no differences in RFS [33]. The authors indicated that 81.4% of patients with tumors under 1.5 cm did not need a second intervention during the follow-up. One of the latest observational studies by Bozzini et al. reported a low upper tract recurrence rate of 19.2% during a median follow-up of 11.7 months after the Tm:YAG ablation of low-grade tumors [36].

### 3.5. Greenlight Laser, Diode Lasers, Thulium-Fiber Laser

Greenlight lasers are derived from the KTP:YAG or LBO:YAG lasers. The wavelength of 532 nm results in strong absorption by hemoglobin. Ex vivo trials have shown deep coagulation and high vaporization effects at high-power settings [37]. These characteristics may make them candidates for vaporization of UTUC, but the significant coagulation depth of the Greenlight laser limits its usability for UTUC treatment, resulting in limited evidence regarding its efficiency [38].

The term “diode laser” refers to the laser firing as generated by a diode source. There are several wavelength variations of these lasers which result in different effects on the tissue. For example, 1470 nm diode lasers show deep coagulation zones–1.30–2.30 mm, which are in the range of the Nd:YAG laser [39,40]. Similar to Nd:YAG, diode lasers are absorbed by water, and the main effects are tissue vaporization and coagulation, but not as deep as Nd:YAG. These make diode lasers a promising tool for the treatment of UTUC, especially for patients who require good intraoperative hemostasis, e.g., those receiving anticoagulant therapy [41]. However, the deep tissue effects of diode lasers and Nd:YAG theoretically might cause ureteral perforation and thus have to be applied carefully for the endoscopic treatment of UTUC. The experience of diode laser (T-1470 LiteTouch™) use for UTUC treatment is described mostly in case reports with promising reports regarding perioperative outcomes [38,42].

The thulium-fiber laser (TFL) is a new laser device that has shown value in the treatment of NMIBC [43]. There are no articles in English that discuss the use of TFL for UTUC treatment. However, TFL might be useful in UTUC treatment settings. Ex vivo trials have shown that TFL makes narrow, clean and deep incisions (2.7 mm) with non-extensive carbonization [24]. In comparison with Ho:YAG, TFL is better in terms of coagulation (up to 0.6 ± 0.2 mm) and is, therefore, better suited for patients on anti-coagulant therapy [24]. However, no separate analysis on the performance of TFL for the treatment of UTUC has yet been reported [44].

### 3.6. Dual Laser Generators

Dual laser generators are also used for treating UTUC and make it possible to combine the advantages of two lasers in one device. However, no combined firing can be expected in this case. Lasers operate through the same surgical fiber, one after the other. This results in it surpassing the coagulation and vaporization ability of Tm:YAG with a carbonization-free cutting of Ho:YAG. In a retrospective study, when used for Retrograde intrarenal surgery (RIRS), the Thulium-Holmium:YAG Duo laser resulted in a low risk of complications (10%, only Clavien-Dindo grade I) and a high kidney preservation rate with a recurrence-free survival (RFS) rate of 69.3 % after a mean follow up of 28.7 months, [7]. Yoshida et al. [45] compared the oncological outcomes of combined Tm:YAG/Ho:YAG setting for tumor ablation with their previous results on the performance of Ho:YAG. They determined that the combined setting had a better 2-year progression-free survival than single Ho:YAG (100% vs. 58.7%, *p* = 0.0197 respectively), better 2-year recurrence-free survival rate (57.1% vs. 41.3%, *p* = 0.072 respectively) and lower salvage RNU rate (0.0% vs. 50%; *p* = 0.009 retrospectively). Also, the rate of grade ≥ 3 complications was lower after combined laser treatment (0.0% vs. 6.7% retrospectively). The successful tumor ablation was achieved in all cases (in Ho:YAG group, it failed in 18.7% of patients). Today, that type of dual laser generator remains popular among urologists for laser ablation treatment of UTUC. For example, Miyake et al. [46] are preparing a prospective study of combined Tm:YAG/Ho:YAG laser impact on 5-year outcomes.

A dual laser generator that contains a neodymium laser (Nd:YAG) and a holmium laser (Ho:YAG) is also a feasible option for the endourological treatment of UTUC and leads to similar short-term oncological results [14,47]. Hubosky et al. presented the results of the combined Nd:YAG/Ho:YAG laser generator in the UTUC treatment [48]. In their study, successful tumor ablation was achieved in 12 out of 15 patients after initial URS. Of those, three patients had ipsilateral tumors at 3, 12 and 177 months, respectively. The study was performed on patients with Lynch syndrome, which might be the reason for the manifestation of a metachronous bladder tumor in six patients within 10 months after surgery. 

In the article by Scotland et al. [49], the outcomes of 63 patients with tumors larger than 2 cm were presented. All of them were treated with Nd:YAG/Ho:YAG laser generator; 57 (90.5%) had tumor recurrence during follow-up, and the mean time to first recurrence was 4.9 months; 12 patients (31.7%) had a disease progression in grade at a median of 26.3 months, and seven (11.1%) patients developed metastatic disease. As for long-term (5 years) outcomes, overall survival was 75%, and cancer-specific survival was 84%; 16 patients underwent subsequent RNU. The same devices were used by the group of Mugiya et al. [50] to estimate the prospects for a bad prognosis after tumor laser ablation. In their trial, which included seven patients, all patients (2/2) with tumor grade two died during the follow-up period—at 22 and 30 months after surgery, respectively; two more patients left the study for other reasons. Each of the remaining three patients underwent 5.3 ureteroscopic procedures on average due to the 2.7 recurrences detected during a mean follow-up period of 33 months. In the other study of the same research group, six patients were treated. The patient with a Grade 2 tumor of 3 cm in diameter died from cancer 22 months after surgery [51]. Also, two patients left the study for other reasons. The remaining three patients showed no evidence of disease at a mean of 12 months of follow-up. 

The study of Keeley et al. [52] included 40 patients treated with Nd:YAG/Ho:YAG dual generators. They found that 15 out of 21 (71%) patients with low-grade tumors (G1/1-2) showed no evidence of disease after surgery; six out of 14 patients (43%) with Grade 2 tumors also stayed tumor-free after surgery; two out of five patients (40%) with Grade 3 tumors were rendered disease-free. However, the authors pointed out that follow-up was too short to assess recurrence rates. The trial by Boorjian et al. [53] produced similar results. In their study, 26 of 38 (68.4%) patients had at least 1 recurrence at a mean follow-up of 37.2 months. The authors found a correlation between abnormal urine cytology and the recurrence rate—16 of the 17 patients (94.1%) with initially abnormal findings experienced at least one recurrence, compared with eight out of 17 patients (47.1%) with an initially negative selective cytology result (*p* = 0.0026). Another study of the same group [54] was proposed to compare the postoperative disease status of patients. In particular, this study would focus on patients who had undergone nephroureterectomy on the basis of positive cytology findings and filling defect on contrast imaging (no ureteroscopy; n = 34) as well as patients who had undergone nephroureterectomy after ureteroscopic biopsy (n = 75) and patients who underwent nephroureterectomy after ureteroscopic biopsy and laser tumor ablation by dual laser generator (n = 12). This study found that 29 out of 34 (85.3%) patients in the “no biopsy” group were disease-free at a mean follow-up of 38.7 months; 61 out of 75 (81.3%) patients who underwent ureteroscopic biopsy before nephroureterectomy had that status when their mean follow-up after nephroureterectomy was 40.1 months; and 10 out of 12 (83.3%) patients who underwent nephroureterectomy after ureteroscopic biopsy and laser tumor ablation were disease free at a mean follow-up of 37.2 months.

The summary of the most relevant publications on the topic is presented in Table 1.

## 4. Discussion

The percentage of patients that are treated endoscopically is on the rise. There are a number of reasons for this: (1) expanding indications for kidney sparing, (2) satisfactory oncological results, and (3) new types of laser devices. However, in all cases, a stringent long-term follow-up (>5 years) is required: urine cytology and cross-sectional urography or endoscopic visualization are a must after nephron-sparing treatment due to the high risk of disease recurrence [1].

The issue of deciding on the most suitable laser for KSS remains unresolved due to the limitations presented in the available studies. Given the mostly retrospective nature of the studies and the rarity of UTUC, the authors often do not indicate the type of laser used for KSS [55,56,57,58]. There are also very limited data comparing different devices in terms of their efficacy and special features. The oncological outcomes, especially the overall and cancer-specific survival presented in most papers, depend on multiple factors other than the device used for the treatment and, therefore, do not serve as adequate benchmarks to compare laser performance. 

In the current paper, we are not focusing on the issue of laser KSS’s safety. The reason for this is there was a lack of data in most of the research articles, focusing instead primarily on oncological outcomes. However, generally speaking, the complication rate for retrograde laser ablation of UTUC is low. In a recent systematic review, Laukhtina et al. estimated the complication rate for retrograde KSS intervention: any grade complications were observed in only 6.6% of patients. Moreover, most of these were minor (Clavien-Dindo I–II) complications [59]. The risk for ureteral structure development was 6.6%. However, the authors noticed that many of the patients who underwent retrograde KSS had had (as is normally the case) a high number of previous interventions (up to 18 procedures per patient).

We should understand better what the surgeon needs/seeks from KSS in UTUC. Unlike the non-muscle invasive bladder cancer resection [60], obtaining the whole UTUC specimen is not so important for tumor staging [61]. What is mostly needed is a tool that allows—after obtaining the biopsy—to completely and safely remove the tumor. The priority in the endoscopic management of UTUC is the safe and complete removal of the tumor. Thus, most of the above-discussed lasers are adequate choices for kidney-sparing surgery in UTUC, provided there is an understanding of their collateral effects on the tissue. In general, the ideal laser should not produce too much carbonization and should not enhance the risk of thermal damage, as lasers, when firing, can be absorbed by hemoglobin. Therefore, the ideal candidates for this kind of surgery are Tm:YAG and Ho:YAG (especially in dual-laser generators) and possibly TFL. Unfortunately, we have no data on the latter regarding its safety and efficacy in clinical settings. Despite this, any laser generator can be useful in this setting, but we should always bear in mind the advantages and disadvantages of the different devices in order to maximize the safety of our patients.

## 5. Conclusions

At the moment, there are no data that definitively show one laser being better than the other in the endoscopic management of UTUC. And so it really comes down to the surgeon’s personal experience and preference when choosing the right device. The expanding indications for minimally invasive KSS treatment of UTUC and the increasing number of centers specializing in laser surgery give us hope that proper well-designed prospective studies with adequate conclusions will be reported in the years to come. Given the rarity of UTUC, such trials will likely rely on multicenter and international collaborations. Nevertheless, we are sure that laser therapy will remain and expand as the energy source of choice in the diagnostic and therapeutic management of UTUC.

## Figures and Tables

**Table 1:** Lasers for the UTUC treatment.

Publication	Year	Number of Participants	Disease Stage	Mean Follow-Up (Months)	Outcomes Measurement	Results
Nd:YAG						
Malloy et al. [20]	1986	6	Clinically localized exophytic renal tumors	14	Recurrence-free rate	6/6
Orihuela et al. [18]	1988	14	Low- and high-grade tumors	19	Recurrence-free rate	6/14
Grossman et al. [17]	1992	8	Imperative or superficial disease with a normal contralateral kidney	21	Recurrence-free rate	2/8
Kaufman et al. [19]	1993	9	Low-grade, low-stage transitional cell carcinoma of the ureter	28	Recurrence-free rate	8/9
Elliott et al. [21].	1996	44	Renal pelvic tumor sizes ranged from 0.4 to 4.0 cm (mean, 1.5) and ureteral tumors from 0.2 to 1.0 cm (mean, 0.5). Pathologic grade 3 or less, Stage T2 or less.	60	Disease-free rate	57%
Elliott et al. [22]	2001	21	8 renal pelvic tumors, 13 ureteral tumors, Stage T1 or less and grade 3 or less, less than 2 cm in the greatest dimension	74	Recurrence-free rate	75%
Niţă et al. [16].	2012	44	Imperative (41 cases) or elective (24 cases)	60	Recurrence-free rate for low-grade tumors for high-grade tumors	36.36% 71.42%
Nd:YAG or Ho:YAG						
Scotland et al. [5]	2020	168	Grade 1–3 tumors	60	Overall survival Cancer-specific survival Recurrence-free survival	80.9 92.6 30
Ho:YAG						
Matsuoka et al. [30]	2003	7 and 20	Patients with a single kidney and low-grade cancer	37 and 33	Tumor-free rate	57% and 95%
Painter et al. [28]	2008	19	Grade 2 or less	24	Progression-free rate	17/19
Cornu et al. [26]	2010	35	15 patients with imperative indications, 20—elective	36	Disease-specific survival Recurrence-free rate	100% 35%
Aboumarzouk et al. [31]	2013	19	Grade 1 or 2, unifocal, less than 1.5 cm	24	Cancer-free rate	13/19
Hoffman et al. [27]	2014	25	Low-grade tumors less than 1.5 cm	26	Bladder recurrence rate	44%
Villa et al. [9]	2018	92	Low- and high-risk cancer	52	Progression-free rate in low-grade tumors in high-grade tumors	75% 52%
Yamane et al. [29]	2022	2	Low-grade tumors	60/84	Recurrence-free rate	100%
Tm:YAG						
Defidio et al. [33]	2011	59	Tumors under 1.5 cm	-	Recurrence-free rate	81.4%
Musi et al. [34]	2018	42	low- and high-grade cancer	26	Recurrence-free rate	81%
Wen et al. [35]	2018	32	low- and high-grade cancer	-	Recurrence-free rate	78.1%
Bozzini et al. [36]	2021	-	low-grade tumors	11.7	Recurrence-free rate	80.8%
Combined Nd:YAG/Ho:YAG						
Keeley et al. [52]	1997	40	Grade 1–3	35.1	Disease-free rate in the grade 1 group in the grade 2 group in the grade 3 group	15/21 6/14 2/5
Mugiya et al. [51]	2003	6	4 p.—Grade 1; 2 p.—Grade 2	14	Recurrence-free rate	3/6
Boorjian et al. [53]	2004	38	17—negative biopsy, 17—abnormal biopsy	37.2	Recurrence-free rate	12/38
Boorjian et al. [54]	2005	12	-	37.2	Disease-free rate	10/12
Mugiya et al. [50]	2006	7	5 p.—Grade 1; 2 p.—Grade 2	32	Recurrence-free rate	0/7
Hubosky et al. [48]	2013	15	patients with Lynch syndrome	10	Successful ablation rate	12/15
Scotland et al. [49]	2018	63	tumors larger than 2 cm	60	Overall survival Cancer-specific survival Recurrence-free rate Progression-free rate	75% 84% 9.5% 68.3%
Combined Tm:YAG/Ho:YAG						
Yoshida et al. [45]	2021	-	-	24	Progression-free survival Recurrence free-rate	100% 57.1%
